# Supplementary material for: X-ray crystallography reveals molecular recognition mechanism for sugar binding in a melibiose transporter MelB
Source: Commun Biol. 2021 Aug 2;4:931. doi: 10.1038/s42003-021-02462-x (PMC8329300; doi:10.1038/s42003-021-02462-x)
Supplement: Supplementary file 2 — Supplementary Information [file 42003_2021_2462_MOESM2_ESM.pdf]

Supplementary Figure

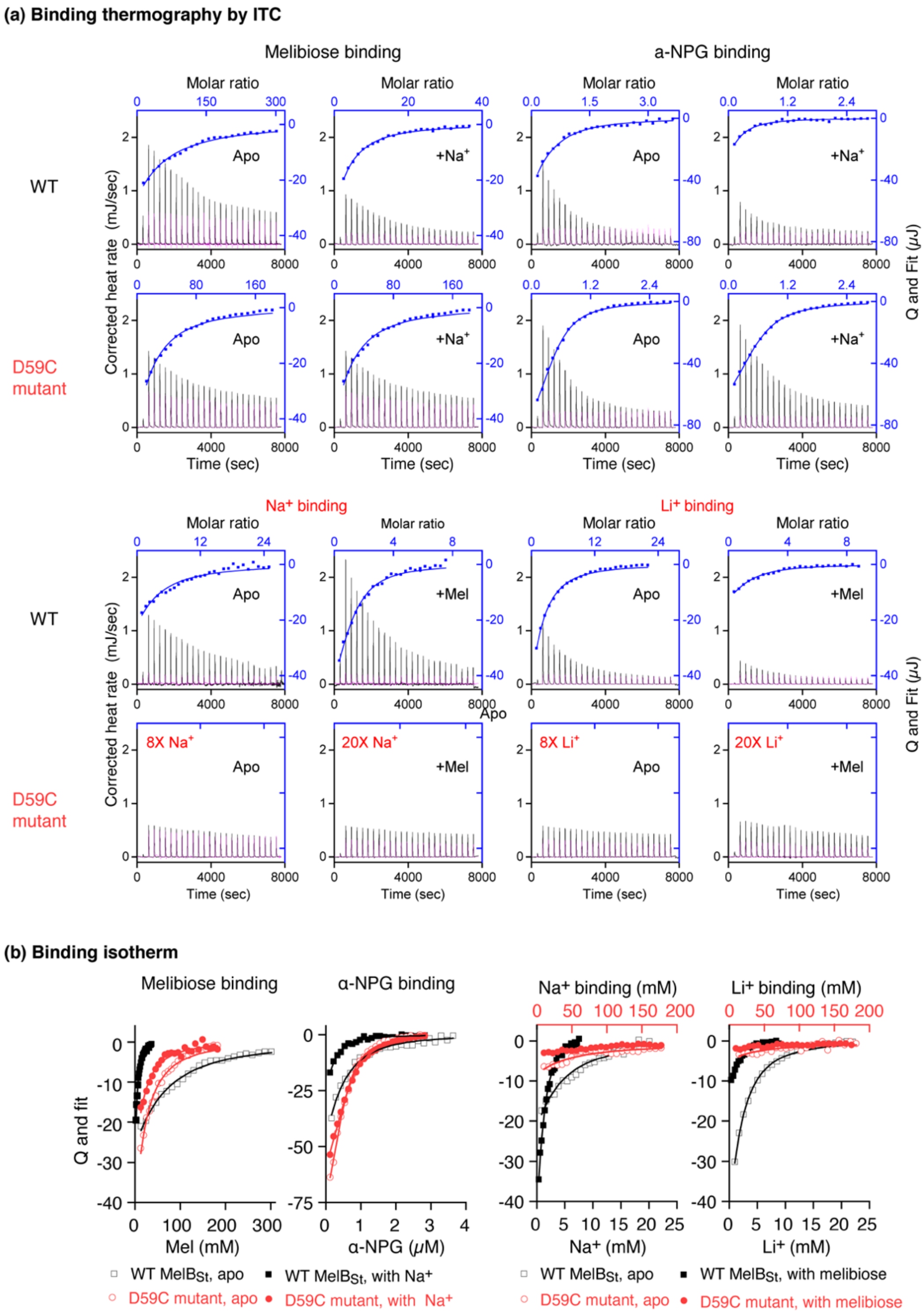

Supplementary Figure 1. ITC measurements. ITC measurements were performed at 25 °C as described in Methods. **(a) Binding thermography.** For all tests, ligands and proteins are buffer-

matched. For melibiose binding measurements, a solution of 80 mM or 10 mM in the absence or presence of NaCl at 100 mM, was injected into the ITC sample cell containing the WT $MelB_{St}$ at 80 μM without or with 100 mM NaCl, respectively. With the D59C mutant, 40 melibiose was used. For α-NPG binding measurements, 0.5 mM or 0.4 mM solution was used to titrate the WT or D59C mutant at 50 μM in the absence or presence or 100 mM NaCl, respectively. For the Na^+ or Li^+ binding measurements, 5 mM or 2 mM solution was used to titrate WT $MelB_{St}$ at 80 μM without or with 50 mM melibiose, respectively; 40 mM solutions was used to titrate the D59C mutant. **(b) binding isotherm**. Curve fit was carried out with a one-site independent-binding model included in the NanoAnalyze software (version 3.6.0). Results are presented in **Table 1**.

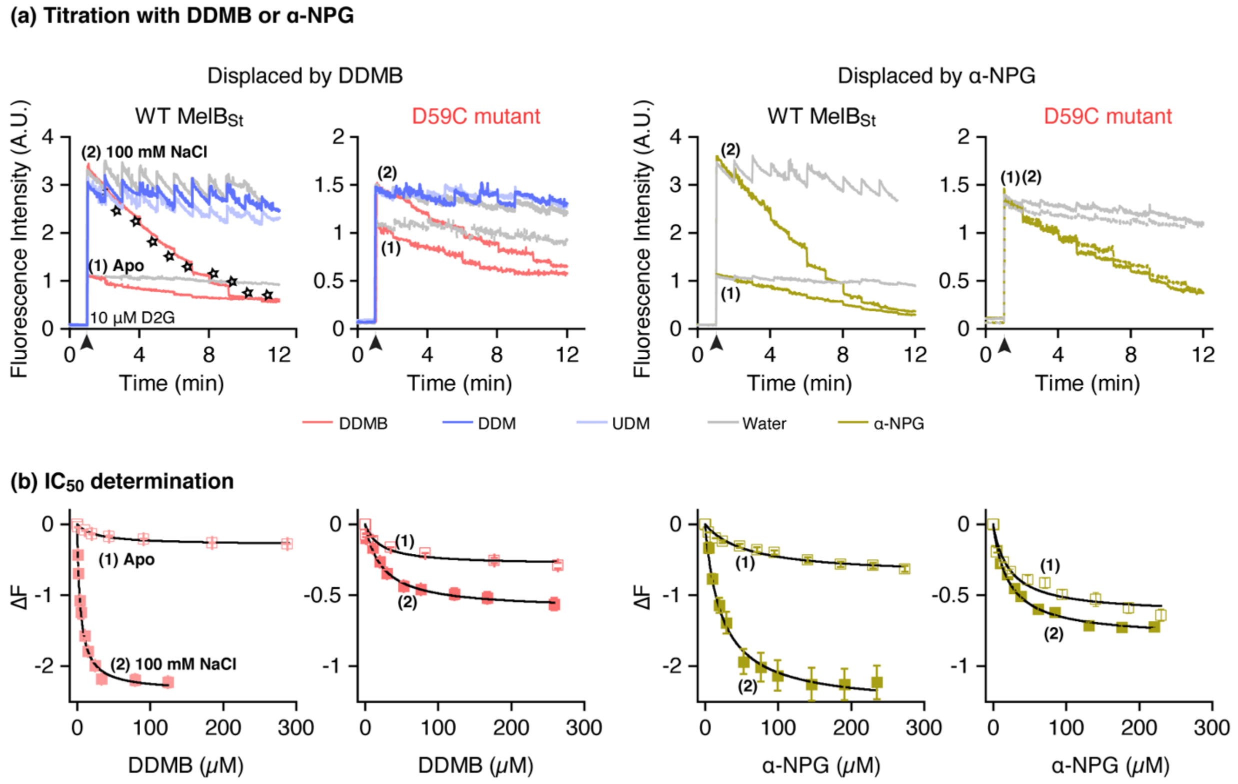

Supplementary Figure 2. Determination of DDMB binding affinity to MelB$_{St}$. (a) Titration with DDMB or α-NPG. MelB$_{St}$ in 20 mM Tris-HCl, pH 7.5, 100 mM CholCl, 10% glycerol, 0.03% UDM without (1) or with 100 mM NaCl (2) were added with D^{2}G at 10 μM at 1-min time point as indicated by triangle symbols on the Trp→dansyl FRET time trace. Starting at the 2-min of time point, DDMB or α-NPG solution was supplemented consecutively at a 1-min interval into MelB$_{St}$ solution till no change in fluorescence intensity reached as indicated in star symbols. DDM and UDM were used as negative controls. 1% DDM or UDM was titrated into the mixture, The accumulated functional concentration for both is less at 300 μM, which is close the DDMB CMC value. At 2-min time point, additions of galactosides were colored in pink, α-NPG in yellow, UDM in blue, DDM in light blue, water in gray traces, respectively. **(b) IC50 determination**. The intensity decrease at each titration were corrected by the dilution effect, and plotted as a function of the accumulated DDMB or α-NPG concentration. The 50% inhibitory concentration (IC$_{50}$) was determined by fitting the data to a hyperbolic function (OriginPro 2020b). Results are presented in **Table 2**.

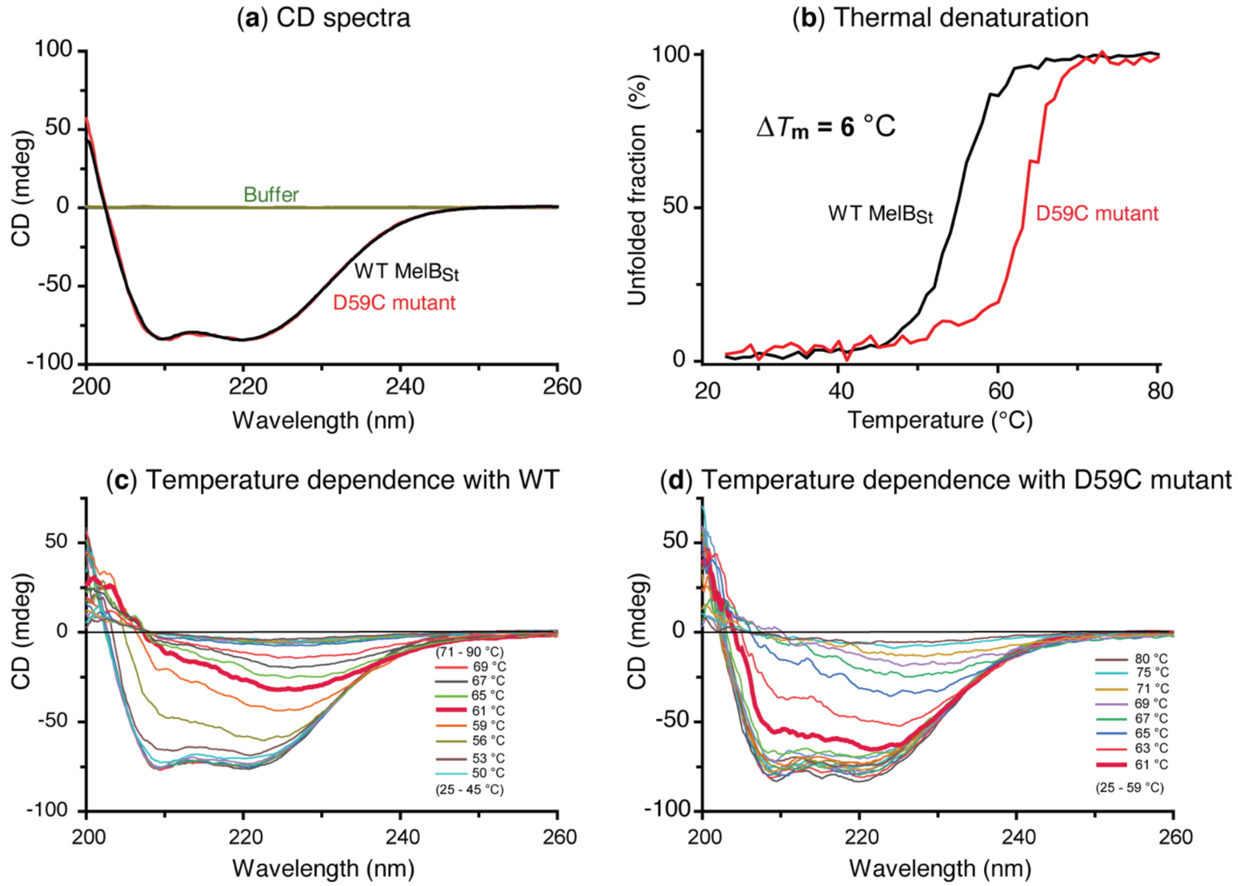

Supplementary Figure 3. CD spectra and T_m determination. $MelB_{St}$ at 10 µM in 10 mM NaPi, pH 7.5, 100 mM NaCl, 10% glycerol, and 0.035% UDM was used for the CD analysis as described in Methods. **(a) CD spectra**. The CD was recorded between 200 – 260 nm for both the WT and D59C mutant $MelB_{St}$ in the absence of sugar. **(b) Thermo-denaturation.** The tests were carried out between 25 to 80 °C in the absence of sugar. The T_m values was determined using the Jasco Thermal Denaturation Multi Analysis Module.

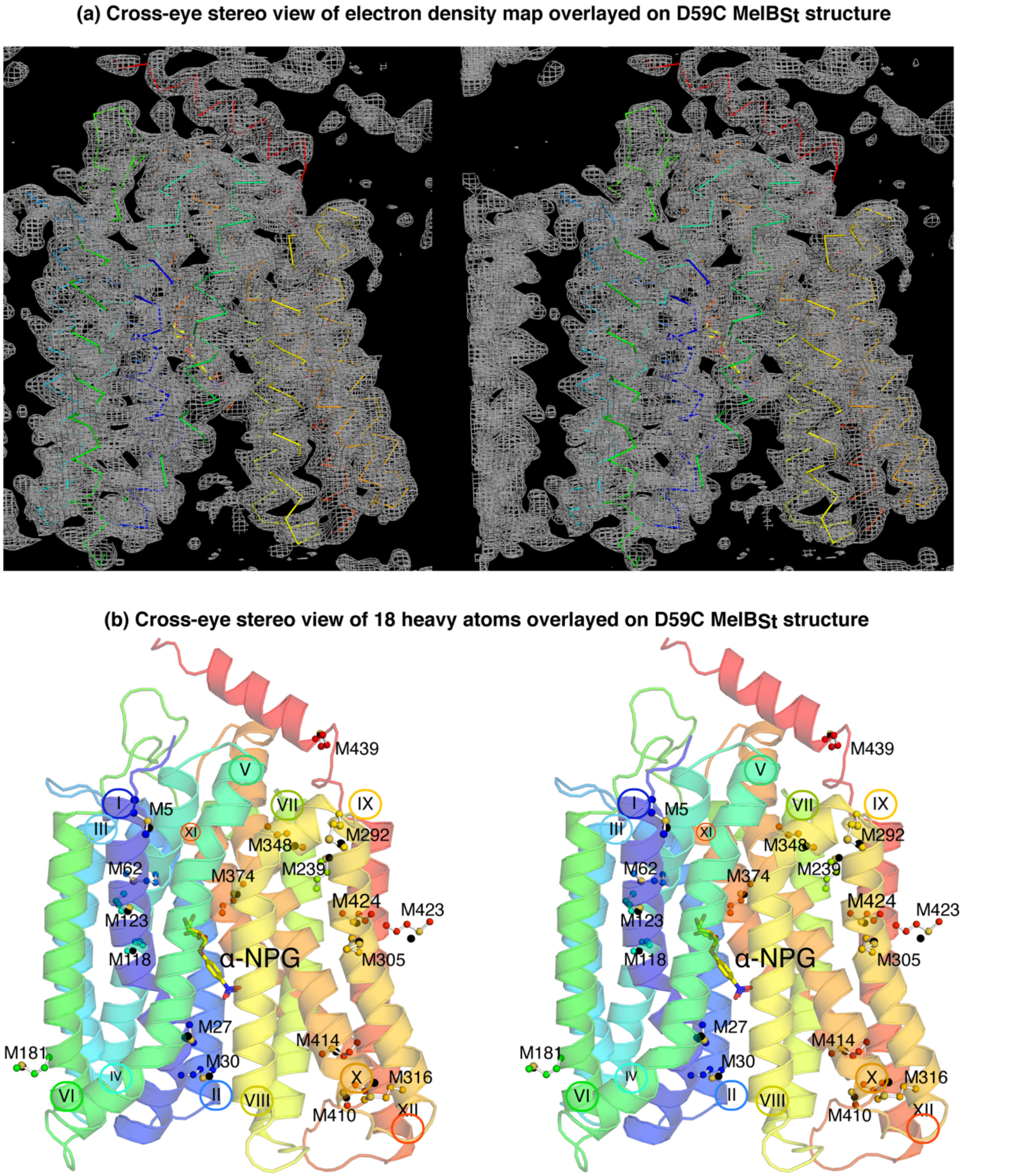

Supplementary Figure 4. Stereo view of electron density maps of D59C MelB$_{St}$ bound with α-NPG. (a) Cross-eye stereo view. The structure for D59C MelB$_{St}$ bound with α-NPG [PDB ID, 7L17] is shown in ribbon representation and overlayed with 2Fo-Fc electron density map contoured at 1.5 σ. Helices V and VIII are placed in the front. α-NPG is shown in sticks. **(b)** Cross-eye stereo view of selenium atoms. A total of 18 selenium atoms identified based on anomalous signal of selMet D59C MelB$_{St}$ bound with DDMB were overlayed with refined structure of D59C MelB$_{St}$ structure bound with α-NPG. The protein is shown in cartoon representation; 18 Met sidechains are shown in stick and ball with sulfur atom colored in yellow and carbon atoms colored matching with individual backbones. Selenium atoms are colored in black. Except for M316 and M423, all other overlayed well with selenium atoms. The bound α-NPG is shown in sticks.

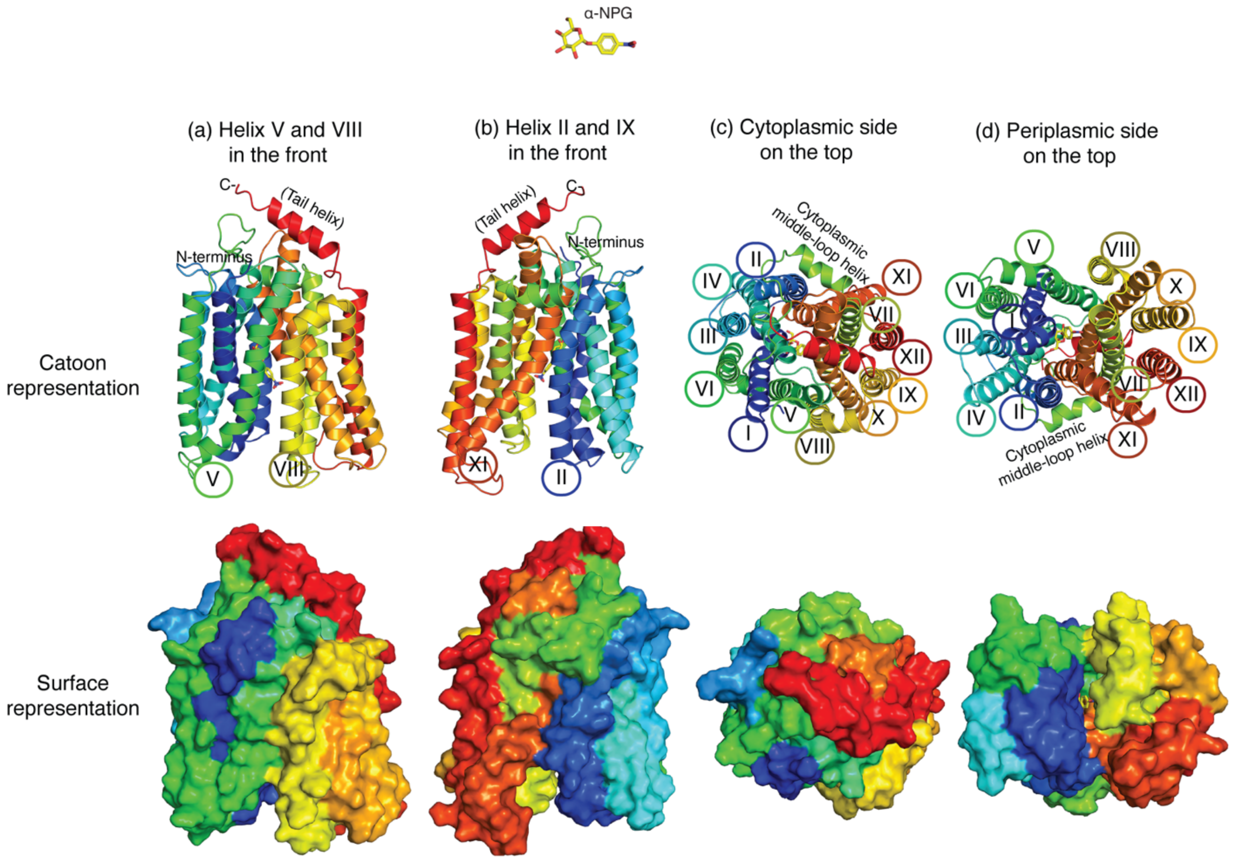

Supplementary Figure 5. Helical packing and overall architecture of MelB$_{St}$. The α-NPG-bound D59C MelB$_{St}$ structure [PDB ID, 7L17] is colored in rainbow spectrum. The bound α-NPG in each panel is colored in yellow. Upper row, helical packing in cartoon representation; bottom row, surface representation. **(a-b) Side view with helices V and VIII in front or II and XI in front, respectively. (c) Viewed from cytoplasmic side. (d) Viewed from periplasmic side.**

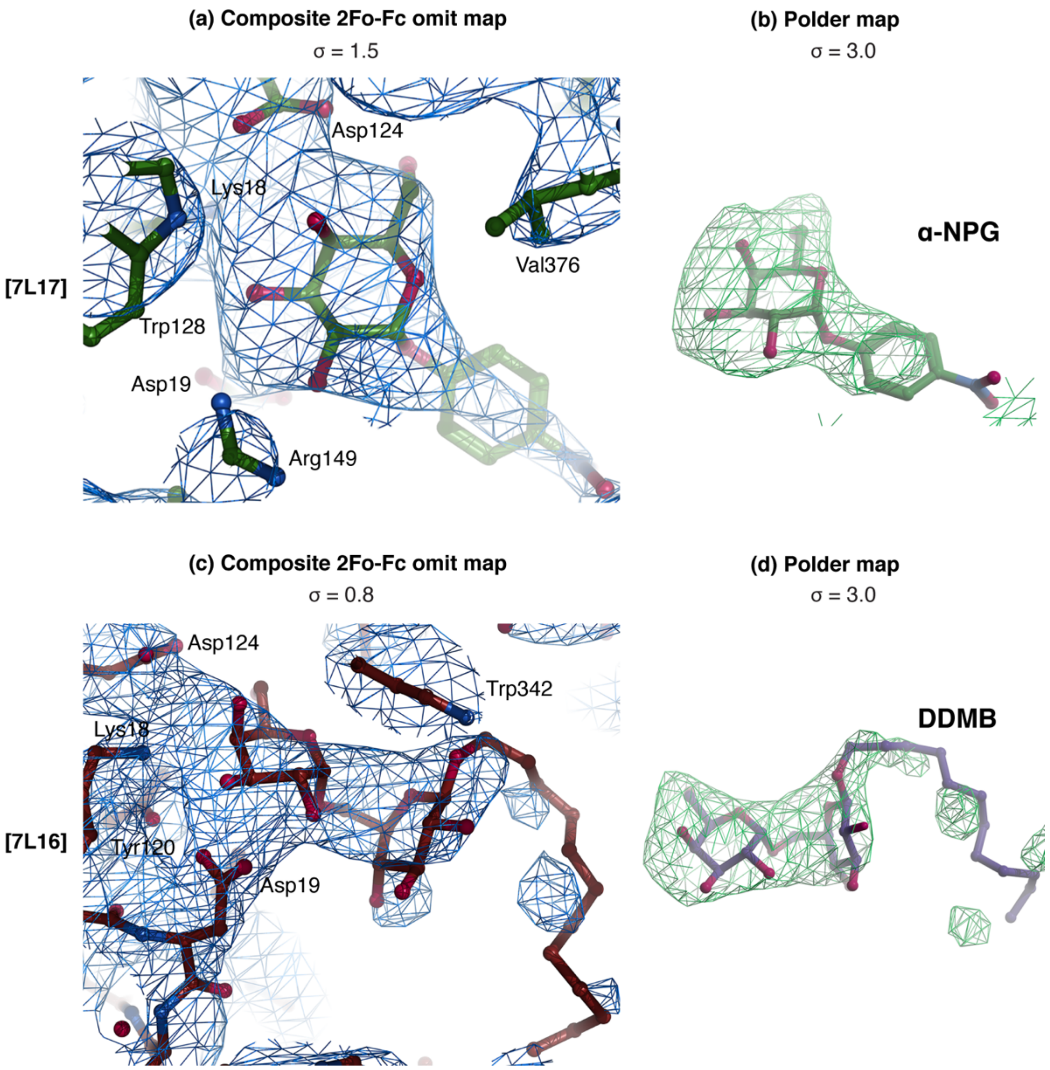

Supplementary Figure 6. Maps. (a, b) The 2Fo-Fc composite omit map and Podler map from the α-NPG-bound D59C MelB$_{St}$ structure [PDB ID, 7L17]. The two maps were calculated in Phenix and contoured to σ of 1.5 or 3.0, respectively, as indicated. **(c, d) The 2Fo-Fc composite omit map and Podler map from the DDMB-bound D59C MelB$_{St}$ structure [PDB ID, 7L16].** The two maps were calculated in Phenix and contoured to σ of 0.8 or 3.0, respectively, as indicated.

(a) Overview of sugar site and cation site of D59C MelB$_{St}$

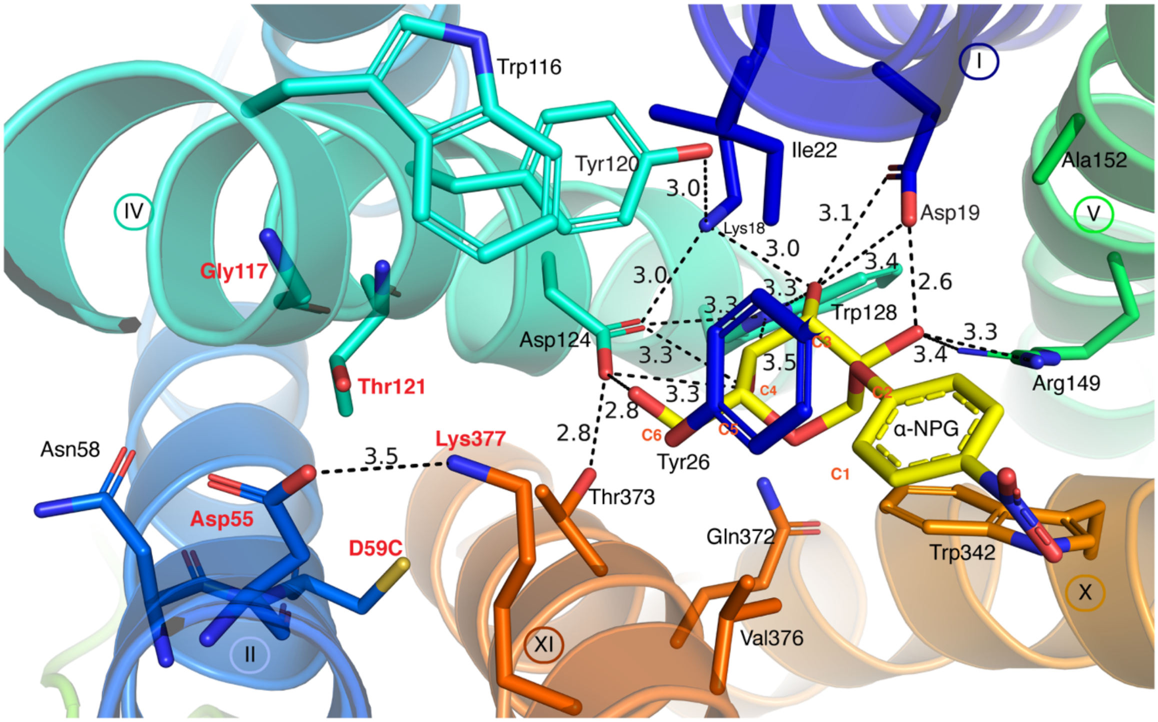

(b) Multiple sequence alignment focused on the active site

		I	II	IV	V	X	XI
		18 19 22 26	55 58 **59**	117 120 121 124 128	149	342	373 376 377
P30878	MELBSt	18-KDFAIGIVY-26	55-DAIND-59	117-GMTYTIMDIPFW-128	149-R	342-W	373-TMVVK-377
Q7VAQ3	Q7VAQ3_PROMA	11-TGLAATQLG-19	48-DAIND-52	110-MTAYTSVNLPFA-121	142-F	336-Y	371-VLIQK-375
Q8R8R3	Q8R8R3_THETN	50-IGIMLEAFG-58	87-FAIFN-91	148-DFLYVLVGLNLA-159	180-Q	356-V	387-GFIIR-391
Q65SP4	Q65SP4_MANSM	20-CNLVFSTMA-28	58-DAVTD-62	119-SLAYTVVNIPLN-130	151-M	335-F	365-GFFVK-369
Q65T75	MELB_MANSM	18-KDFAIHIVY-26	55-DAVND-59	117-GMTYTLMDIPFW-128	149-R	342-W	374-TMVVK-379
O07366	MELB_KLEAE	18-KDFAIGIVY-26	55-DAIAD-59	117-GFTYTIMDVPFW-128	149-R	342-W	373-TLVVK-377
Q02581	MELB_KLEPN	18-KDFAIGIVY-26	55-DAIAD-59	117-GFTYTIMDVPFW-128	149-R	342-W	373-TLVVK-377
P02921	MELB_ECOLI	18-KDFAIGIVY-26	55-DAIND-59	117-GMTYTIMDIPFW-128	149-R	342-W	373-TMVVK-377
Q83PA7	MELB_SHIFL	14-KDFAIGIVY-22	51-DAIND-55	113-GMTYTIMDIPFW-124	149-R	342-W	369-TMVVK-373
Q8XDU0	MELB_ECO57	18-KDFAIGIVY-26	55-DAIND-59	117-GMTYTIMDIPFW-128	149-R	342-W	373-TMVVK-377
Q9KWG6	MELB_CITF	18-KDFAIGIVY-26	55-NAFND-59	116-GMTYTIMDIPFW-127	148-R	341-W	372-TMVVK-376
Q8Z1N9	MELB_SALTI	18-KDFAIGIVY-26	55-DAIND-59	117-GMTYTIMDIPFW-128	149-R	342-W	373-TMVVK-377
Q57GS8	MELB_SALCH	18-KDFAIGIVY-26	55-DAIND-59	117-GMTYTIMDIPFW-128	149-R	342-W	373-TMVVK-377
Q5PJ19	MELB_SALPA	18-KDFAIGIVY-26	55-DAIND-59	117-GMTYTIMDIPFW-128	149-R	342-W	373-TMVVK-377
Q14CX5	MFSD13A_HUMAN	26-TTILHNVFL-34	63-NSLND-67	135-DGFLTLVDLHHH-146	167-S	375-C	408-ALVTK-412
Q8NA29	MFSD2A_2_HUMAN	56-YQVTGCALG-64	93-DAITD-97	155-ETMVTCFHVPYS-166	187-M	399-F	432-VFFTK-436
A6NFX1	MFSD2B_HUMAN	54-NQIASSATA-62	91-GAAAD-95	153-QALATFFQVPYT-164	185-M	386-L	419-VFFTK-423

Supplementary Figure 7. Cation and sugar sites. (a) An overall view of co-substrate binding sites. The specific determinant pocket is closely connected with the proposed cation site as labeled in red. Helices are shown in a cartoon representation and labeled in Roman numerals; α-NPG is colored in yellow with oxygen in red and nitrogen in blue. **(b) Alignment of selected MelB homologues.** MelB$_{St}$ is aligned with other 13 bacterial orthologues that are melibiose transporters or function unknown transporters, or with three selected human transporters, respectively. Protein sequence access identifications are presented. Selected regions containing the positions involved in sugar binding and cation site are isolated and presented. The transmembrane locations are indicated according the MelB$_{St}$ structure. Yellow color high-lights the identical positions that in

MelB is in the galactosyl moiety-binding site (18, 19, 22, 26,124, 128,129, 342, 376). Except for Ile22, Tyr26, and Val376 with hydrophobic contacts, all are engaged in one to three H-bonding interaction(s) with galactosyl moiety. Pale yellow highlights the identical positions involved in the H-bonding network with no direct contact with galactoside in MelB. Color in cyan highlights the identical positions involved in cation binding in MelB. The sequence for MELB_ECO57 was renumbered by adding MSIS- at the N-terminus. It is noteworthy that "MSIS-" sequence for most MelB was missed at earlier time, which might cause a confusion; such as, Asp55 and Asp59 were named as Asp51 and Asp55, respectively.
