# Supplementary material for: X-ray crystallography reveals molecular recognition mechanism for sugar binding in a melibiose transporter MelB
Source: Commun Biol. 2021 Aug 2;4:931. doi: 10.1038/s42003-021-02462-x (PMC8329300; doi:10.1038/s42003-021-02462-x)
Supplement: Supplementary file 3 — Description of Additional Supplementary Files [file 42003_2021_2462_MOESM3_ESM.pdf]

Description of Additional Supplementary Files

File name: Supplementary Data 1

Description: This Excel file contains all original data used directly for generating the Figure 1 a (melibiose transport) and b (FRET).
